# Where National Medicines Policies Have Taken Us With Patient Involvement and Health Technology Assessment in Africa

**DOI:** 10.3389/fmedt.2022.810456

**Published:** 2022-02-24

**Authors:** Kawaldip Sehmi, Janet L. Wale

**Affiliations:** ^1^International Alliance of Patients' Organizations, London, United Kingdom; ^2^HTAi Patient and Citizen Involvement Interest Group (PCIG), Brunswick, VIC, Australia

**Keywords:** patient involvement, civil society, health technology assessment, national medicines policies, regulation, globalization, low and middle-income countries

## Abstract

The Covid-19 pandemic has highlighted global knowledge about, but lack of equitable access to, life-changing medicines, and other innovative medical products by populations in African low and middle income countries. The World Health Organization (WHO) and other international non-profit foundations and organizations are constantly striving to address inequity. In the 1970s, WHO initiated a regularly updated essential medicines list, together with the concept of national medicines policies (NMPs) to ensure access and availability, affordability, rational, and effective use of medicines which are considered essential in addressing predominant population health issues and disease burden. We studied the NMPs of Ghana, South Africa, Uganda and Zimbabwe to highlight some of the important issues that these countries experience in the safe and effective use of medical products. Thailand is an example of how health technology assessment (HTA) can provide a country with an internationally supported, clearly defined and transparent process to broaden access to medicines and services. These medical services can add considerable value in accordance with local values and priorities. Involvement of civil society adds democratic legitimacy to such processes. Community health workers and patient advocacy groups are important in raising awareness and knowledge of safety issues and the effective use of quality medicines. They can apply pressure for increased funding to improve access to healthcare. Medicines and services that contribute to supported self-care are of benefit in any setting. Joint efforts across African countries such as with the African Medicines Agency are important in addressing some of the major health issues.

## Working to Achieve Access to High Quality Medicines and Rational Prescribing in Low and Middle Income Countries

In April 2020, >3 months into the Covid-19 pandemic, the World Health Organization (WHO) together with a number of nations launched a cross-discipline partnership to enable resource and knowledge-sharing. The Access to COVID-19 Tools Accelerator included a COVID-19 Vaccine Global Access (COVAX) pillar. Its aim was to rapidly scale up the delivery of vaccines to address high-risk target groups through a scheme of fair distribution (Accelerator) (1). Over time, people without access to resources such as strong health systems, health workers, medicines, and vaccines have become the majority of those who develop Covid-19 infections and die. One publication reported that by late June 2021, 46% of people in high income countries had received at least one dose of Covid-19 vaccine compared with 20% in middle income countries and only 0.9% in low income countries (2). Equitable vaccine distribution was clearly not happening. Broadened vaccine development and approval; scaling up of manufacturing; streamlining shipment, storage, and distribution; and building vaccine confidence were called for. Production and the supply chain was recognized as a barrier to access by high-risk populations and to global vaccination (3).

The 1995 Trade Related Aspects of Intellectual Property Rights (TRIPS) Agreement was set up to provide minimum protection standards for intellectual property, which included pharmaceutical products and vaccines (4). South Africa and India submitted a proposal to the World Trade Organization (WTO), in October 2020, to allow licensing of Covid-19 health products and technologies under the TRIPS agreement (2). They and other countries would then be able to produce Covid-19 medical goods locally and effectively import or export them. India, Egypt, and Thailand were already under license to manufacture viral vector or mRNA-based Covid-19 vaccines. With TRIPS any compulsory licensing arrangements are restricted to domestic purposes only. In order to broaden availability of vaccines, WHO set up an mRNA technology transfer hub in April 2021 to provide the support needed for manufacturers in low and middle income countries (LMICs), and South Africa was selected as the first hub (2).

India has over the years aimed for “abundant availability on a continuous basis, at reasonable prices, of essential, lifesaving and prophylactic medicines of good quality” through local production, generic products, and by managing its tariffs and taxes (5). Standards were benchmarked and harmonized with international standards and practices to ensure high-quality, safe and efficacious pharmaceuticals and to enable growth of an export industry.

Inequities in access to Covid-19 vaccines are simply the latest manifestation of a longstanding problem for Africa and other LMICs. As the prices of medicines and vaccines continue to rise worldwide (6), many LMIC populations are unable to access essential products and are dependent on donations which are often inferior in quality and effectiveness. The WHO has been a key player in more equitable, affordable accessibility to health technologies. Its activities include developing an essential medicines list to prioritize access to and availability of pharmaceutical products, and their rational use (7). The WHO Model List of Essential Medicines (EML) is a list of the medications considered to be most effective and safe in meeting the most important needs in a health system (7). The first list was published by WHO in 1977, and the list is updated every 2 years. Core items are judged to provide the most cost-effective options that require few additional healthcare resources. Other items require infrastructure such as trained healthcare providers, diagnostic equipment, or have a lower cost–benefit ratio. A separate list was created for children in 2007 (6, 7).

Regulating the products entering a country, efficient procurement and ensuring safe and effective distribution are key elements that may be inadequate because of limited funding and lack of a skilled workforce. In response to defined needs, WHO developed and promoted the concept of national medicines policies (NMPs) (8). Of the many WHO programs that support African and other LMICs in their ability to provide medicines to their populations, supporting development of NMPs is one such program (8, 9).

In this perspective we describe how LMICs have used their NMPs to provide essential medicines, improve the safety and quality of medicines, extend their EMLs, and apply rational use of medicines. The introduction of health technology assessment (HTA) processes and the African Medicines Agency are seen as opportunities to broaden discussions on the value of medicines and other technologies and for more unified action to ensure the availability of quality medicines for a wide range of diseases as countries commit to achieving universal health coverage (UHC) by 2030 (10, 11). Each country has its own healthcare challenges that may relate for example to the national political situation, economics and existing legislation. In LMICs access to medicines can be a major issue for the leading politicians and is dependent on the political values of the government, the level of spending on medicines and economic development, and commitment to providing their population with medicines (12).

## National Medicines Policies (NMPs) and Their Role in Promoting Access to Affordable Medicines and Rational Prescribing

### NMPs and Their Goals

Medicines are an important part of healthcare, and the cost of medicines is a key driver of cost of healthcare in African countries. The concept of a NMP was first introduced by the WHO at the 28th World Health Assembly in 1975. The NMP is presented and printed as an official government statement with a framework setting goals and guidance for action (8). The understanding is that for LMICs priority is given to a limited number of carefully selected medicines based on the overall needs of the population. The use of these medicines is supported by agreed clinical guidelines, better supply and procurement, more rational prescribing, and lower costs (9).

### Focus on NMPs for Ghana, South Africa, Uganda, Zimbabwe

We studied NMPs from Ghana (2017), South Africa (1995), Uganda (2015), and Zimbabwe (2011) to gain an understanding of their policies (Tables 1, 2) and the issues they address (13–16).

**Table 1:** National Medicines Policies (NMPs) studied, together with their stated objectives.

**Ghana 2017 (13)**
To bridge equity gaps in geographic access to health services, ensure sustainable funding for healthcare delivery, improve efficiency in governance and management, strengthen prevention and control of communicable and non-communicable diseases, and improve quality of health services including mental health.
**South Africa 1995 (14)**
To ensure the safety, efficacy and quality of drugs; good dispensing and prescribing practices; rational use of drugs by prescribers, dispensers and patients through provision of necessary training, education and information; and to promote the concept of individual responsibility for health, preventive care and informed decision making.
**Uganda 2015 (15)**
To ensure availability and access to affordable drugs to meet the currently recognized needs of the majority of the population.
To provide objective, relevant, and practical information to health workers, patients and the general public.
**Zimbabwe 2011 (16)**
To improve (within the available resources) the health of the majority of the population by treating, curing, reducing or preventing diseases and disorders of health. Equitable availability, accessibility, and affordability of essential medicines, especially to the vulnerable segments of the population, with a focus on priority health problems and, rational use of medicines by health professionals and consumers.

**Table 2:** Issues addressed by national medicines policies in the selected African countries.

The main aim of a National Medicines Policy in the selected African countries is to make available and accessible medicines with the required clinical effectiveness, safety, and quality for evidence based use, and that those provided are cost-effective in their therapeutic group and appropriate to that country
- To have a healthy and productive population that can reproduce safely
**Selection of essential medicines and health technologies**
- Use of an essential medicines list held by government
- To meet the currently recognized needs of the majority of the population
- Standard Treatment Guidelines (STG) as part of the Essential Medicines List
- Reference guidance
**Economic objectives**
- National health policy in alignment with national development
- Lower the cost of drugs (in private and public sectors) through local production
- Support development of local pharmaceutical industry
- Cooperation with regional and international agencies
**Pharmaceutical legislation and regulations**
- Registration of drugs and supplies
- Registration of practitioners
- Licensing of premises; inspections
- Appropriate legislation and regulation on medicines and medical supplies
- Regulatory standards and specifications
- Quality assurance and control
- Post-marketing surveillance, monitoring of adverse medicine reactions (Zimbabwe)
- Advertising, provision of information
- Tariffs and taxes
**Governance**
- Good governance, transparency, and accountability of the pharmaceutical sector
- Risk management
- Organization, management, co-ordination, evaluation of the National Medicines Policy
**Quality assurance**
- Good drug quality control
- Monitor the quality of medical products in circulation and quality defects
**Drug funding**
- Allocation of funds to the public sector so that required essential drugs are continuously available
**Drug pricing**
- Rationalization of pricing structure
- Use of generic drugs
**Appropriate selection of medicines**
To provide quality, safety, efficacy and stable dosage forms. Improve the understanding by health workers, patients and the public on essential drugs (Uganda)
**Local manufacture of drugs**
Incentivize local production of (essential) medicines
**Ensure information on current needs for medicines and the supply situation**
- Ensure uninterrupted supply of medicines
- Avoid wastage and drug expiry caused by over–estimation of requirements, and procurement of low–quality or short shelf–life drugs
**Drug supply and availability of medicines through procurement, distribution, storage**
- Procurement (including donations)—capacity, skills, and experience at all levels of the health system
- Storage and inventory control
- Distribution—quality maintained up to the point of use. Develop procurement so that a system is established
- Optimize utilization of available funding
- Develop trust of the public, donors and all other interested parties in the credibility and validity of the medical supplies management system
- Avoid drug leakages, absence of proper stock management information, and poor storage conditions
**Rational use of drugs**
- Promote rational prescribing, dispensing and use of medicines, by all health personnel
- Ensure that health workers and the general public have access to accurate, up-to-date, unbiased, relevant information on medicines and their use
- Support informed and appropriate use of medicines by the community
- Adhere to ethical criteria for medicines advertising and promotion
- Improve understanding on the place of medicines in a person's treatment
- Ethical procedures in handling medicines, including over-the-counter
- Practical and relevant information on the correct use and storage of medicines
**Use of medicines, information**
- Only obtain medical supplies from suppliers who have acceptable quality standards and procedures
- Promptly address and resolve the quality concerns of health professionals and consumers
- Patient safety
**Disposal of expired or otherwise unwanted drugs and medical supplies**
- Safely dispose of expired and unwanted medicines and related health technologies
- Reduce loss, wastage, and hazards from poor practices throughout the supply chain
**Human resources development**
- Training, recruitment, retention, and development of well-trained health workers at all levels of the health system
- Set and maintain high standards and efficiency in medicine management and handling
- Improve local pharmaceutical technical capacity by training staff in production, quality assurance and Good Manufacturing Practice
**Research and development**
- Operational and Technical Research and development
- On the National Drug Policy
**Global trade**
- Export locally manufactured medicines and vaccines
**Technical cooperation with other countries and international agencies**
**Health technology assessments**
- Collaborate with other HTA groups regionally and globally, to contextualize existing knowledge when available
- Implementation
- Transferability
- Transparency
**Emerging diseases and pharmaceuticals**
- Collaborate with the relevant international organizations to mobilize resources
- Support and fund the research, development and local manufacture of needed products

Uganda (15) sees the objective of its NMP as “contributing to the attainment of a good standard of health by the population, to meet the currently recognized needs of the majority of the population.” The Zimbabwe NMP (16) sets out to “improve, within available resources, the health of the majority of the population by treating, curing, reducing or preventing diseases and disorders of health” (Zimbabwe). Some NMPs focus on a country's priority health problems and the rational use of medicines by both health professionals and consumers (Uganda, Zimbabwe). Good prescribing and dispensing practices; rational use of drugs by prescribers, dispensers, and patients, by providing necessary training, education, and information; and promotion of the concept of individual responsibility for health, preventive care, and informed decision-making (South Africa) are all pertinent elements.

### How NMPs Promote Access to Medicines and Rational Prescribing

The NMPs place an emphasis on developing expertise and human resources in all medicine-handling activities to support successful implementation of the NMP policies (Ghana, South Africa). Training of health workers, including in under-served regions, with sufficient numbers of pharmacy personnel (pharmacists, pharmacy technicians, and dispensing assistants) are specified (Uganda, Zimbabwe). Rational use of medicines is described by WHO as where “patients receive medications appropriate to their clinical needs, in doses that meet their own individual requirements, for an adequate period of time, and at the lowest cost to them and their community” (17).

### Good-Quality, Safe, and Effective Medications

A key component of a NMP is to ensure the safety, efficacy, and quality of medicines, where an emphasis is placed on procurement of generic medicines and the promotion of their use as a means of reducing costs (South Africa, Uganda, Zimbabwe). The Ministry of Health in Ghana with support from development partners centrally procured a number of products in an effort to improve quality. Supply through donations and development partners is a recognized source of medicines in Africa (Ghana), for example in the treatment of tuberculosis, HIV/AIDS, malaria, and in providing Ebola vaccines.

Good governance, management, transparency and accountability, and risk management are important in the procurement of medicines (Ghana, Zimbabwe). For medicines to be continually available and of good quality, a number of factors need to come together including supply to the country, procurement in the public sector, distribution, storage, and inventory control. Steps also need to be taken to prevent theft and waste, and for the safe disposal of unwanted or out-of-date medicines (8). Inferior and fraudulent medicines are a problem, and lead to considerable waste.

Quality control and regulatory procedures need to be strengthened and enforced. The regulatory authority is the agency responsible for developing and implementing much of the legislation, regulations, standards, and specifications on medicines that ensure their quality, safety, and efficacy; together with accurate product information, advertising, and promotion materials (Zimbabwe). Specified standards and mechanisms for manufacturing practices, inspection and law enforcement; registration of medicines and supplies, registration/licensing of practitioners and premises; and inspections are required (South Africa) so that only authorized medicinal products are in circulation (Zimbabwe). Post-marketing surveillance and systems for reporting adverse drug reactions and quality defects have been introduced (Uganda, Zimbabwe).

Importantly, a strong public awareness is needed on appropriate handling and use of drugs and the associated hazards when these are not regulated, as well as the need for effective enforcement and strengthening of regulatory controls (Uganda). Substandard, ineffective or defective vaccines and medicines can enter a country as “gifts,” as has happened during the Covid-19 pandemic (18).

### Enabling Local Manufacture

Essential medicines have been the target for local manufacture, to promote national self-sufficiency in their production (Ghana, South Africa, Uganda, Zimbabwe). Addressing tariffs and taxes to manage prices is one important aspect, as well as establishing regulatory processes.

### Data Collection

NMPs identify the need for greater data collection of sufficient quality for measuring and monitoring the burden of disease, need for services, the effectiveness of healthcare, and medicines and technologies and how they are used. For example, active monitoring and correction to fit with treatment guidelines is needed for prescribing behaviors (Ghana, South Africa, Uganda, Zimbabwe); hospital and district drug and therapeutic committees can provide guidelines and institute a feedback system (Uganda); and data can be used to inform the prescribing and dispensing of antibiotics to reduce resistant microorganisms (19). Data on disease burden and infrastructure can also be used to inform health policy.

## Health Technology Assessment and National Medicines Policies

### What Is Health Technology Assessment (HTA)

HTA is a multidisciplinary process that gathers information about the medical, social, economic, and ethical issues related to the use of a health technology (20, 21). Value judgements are made through multi-stakeholder appraisal committees as these are most likely to pick up unintended consequences of a technology. Each country can make its own philosophical decisions with a societal objective about the healthcare it provides (22). Civil society has an important place in HTA processes, as recognized by INAHTA, the International Network of Agencies for Health Technology Assessment (23) and as argued by Wale et al. (24). From a patient perspective, patients have a right to participate in the planning and delivery of their healthcare, where HTA determines the health services, procedures, and technologies available to them; as a way to build trust in the health system, add value to patients, and center on evidentiary contributions that patients can provide (24). From a methodological perspective, patients may be able to help HTA methodologies evolve, for example by seeking clinical trial outcomes that matter to patients, looking for different clinical trial designs, involving broader groups of people, and in following real world evidence (25).

### Why HTA Is Important

Priority setting and funding are recognized as being value-laden with many factors, or criteria, of importance that extend beyond determination of cost-effectiveness. Because the various stakeholders have different priorities, evidence-informed deliberative processes as institutionalized in HTAs can provide transparency and the space to reflect and learn about the different societal values in the local context (26).

For priority setting to be fair, just distribution through a fair and accountable process is called for. In the African situation, the right to health can be seen as an obligation to be realized over time with dependency on resource availability. National strategies and plans of action are then based on the burden of disease across the entire population and obtained through a legitimate, participatory process. Transparency in prioritization is important, so that civil society and health planners can then advocate for accountability, additional resources, and for delivery of high priority services that provide considerable value (27).

### How NMPs Can Promote HTA

HTA can provide a mechanism for transparent processes for evidence-informed assessment of the value of a medicine or technology leading to managed access to, distribution and rational use with informed reimbursement decisions specific to a country and its priorities in universal coverage of healthcare (20, 26). Good data collection and commitment of government to funding are needed.

The NMPs follow UHC principles as part of the United Nations health-related Sustainable Development Goals (SDG 3), where policy goals are to broaden health coverage to wider population groups; improve financial risk protection; and expand the types of health services people receive (11). Ensuring availability and access to affordable essential drugs in all parts of the country (Uganda), especially to the vulnerable segments of the population (Zimbabwe) is an important aspect of UHC. The first consideration of an NMP is basic essential medicines for all people, before addressing expensive medicines that benefit only a small proportion of the population. This priority is set above “the right to healthcare.” Selection of medicines can also take into account the differing training and skills of the prescribers who are working at different levels of healthcare (see Ghana). These factors, as contained within NMPs, are therefore important to have in place for the fair and equitable introduction of HTA processes.

In 2003, Ghana became the first Sub-Saharan African nation to introduce a tax-funded National Health Insurance Scheme (NHIS). Ghana went on to include the concept of HTA within its 2017 NMP (13).

## Case Studies for Introduction of HTA Processes

### Ghana—Case Study of HTA Using External Resources

In its NMP of 2017, the Republic of Ghana Ministry of Health recognized the potential of HTA to assist in identifying cost-effective health technologies for diagnosis, prevention, and treatment of health conditions. Use of HTA could provide a transparent process for evidence-informed assessment of the value of a medicine or technology and support reimbursement decisions (13). In 2021, Ghana launched its first strategy for HTA. This strategy extends from capacity development, topic selection, and methods guidelines to strategies for implementing HTA findings and assessing impact. It is linked by a strong governance framework (28). Over the last 10 years, the Center for Global Development in Europe (iDSI) built strong government and academic partnerships in Ghana to support government decision-making capacity so it could move “beyond aid” with long-term financial sustainability. The Norwegian Institute of Public Health joined the iDSI network in 2018, to further support this work (29). HTA has already demonstrated its value, for example in changing the formulation of amoxicillin, assessing the COVID-19 Vaccination Plan, and determining the cost-effectiveness of treatments for newly diagnosed hypertension (28). The principles of rational use of medicines are strongly evident in these activities, as promoted by WHO (17).

### Case Study of Thailand as a LMIC That Has Successfully Developed Its Own HTA Program

The concept of incorporating HTA into health policy is relatively new to Africa. A well-documented internationally shared example of successfully initiating HTA processes in a middle income country has been set by Thailand (30). Thailand had well-trained medical practitioners through international partnerships, good healthcare infrastructure, and a national insurance scheme (established in 2001) to provide UHC (31). HTA provided a defined process in coverage decisions for high-cost medicines in the National List of Essential Medicines and to expand the Universal Coverage Scheme (UCS) benefits package. This was in response to increasing public expectations for access to more expensive medical services. Medicines reimbursement was important not just to provide essential medicines and ensure cost-containment, but to reimburse innovative, expensive medicines, and procedures that would add considerable value to healthcare. The HTA processes and methods were developed and adjusted over time. The first research projects, in 2000 and 2004, were joint programs coordinated and funded by Thai government organizations and international funding agencies. Burden of disease studies and priority setting were addressed (e.g., for HIV and rotavirus vaccinations, mental health, cardiovascular disease, diabetes, and road traffic injuries) (30). The government then went on to set up research bodies without international involvement. An HTA unit was established in 2002 focusing on standards of care and quality improvement in Thai top hospitals. In 2007, the unit became the Institute of Medical Research and Technology Assessment and was important for the development of clinical practice guidelines together with economic evaluation. In the same year, the Health Intervention and Technology Assessment Programme (HITAP) was established under the Thai Health Promotion Foundation. HITAP developed HTA and economic evaluation guidelines based on an extensive assessment of existing HTA processes in other countries (Australia, Canada, Denmark, Norway, Hungary, England, and Wales) (32). In Thailand, civil society groups, patient organizations and lay people (from the National Health Assembly) played a role in the prioritization and assessment of proposed health services to be reimbursed by the UCS. For example, in the introduction of innovative renal technologies and in high-cost cancer medications (30).

## Consumer Engagement in LMICs

### Importance of Consumer Engagement in NMPs and HTA—Patient Safety and Advocacy

The highlighted NMPs emphasize the importance of information on medicines for patients and the public, where “objective, relevant and practical information is important to health workers, patients and the general public.” This is to enable improvements in the understanding of the place of medicines in healthcare. The NMPs stress that it is important everyone involved in over-the-counter sales know to handle medicines ethically and that they receive practical and relevant information on the correct use and storage of medicines, for safe, rational and effective use (Uganda, Zimbabwe).

Patient safety has received particular attention in WHO programs and the World Alliance for Patient Safety was launched in 2004. The Patients for Patient Safety global network was created in response to an initial WHO Patients for Patient Safety workshop in London in the following year (33). At this workshop, patients and professionals from 20 different countries including Africa were brought together to create a common vision, guiding principles, and commitment to positive engagement.

The International Alliance for Patient Organizations (IAPO) supports member patient advocacy groups in Africa (34) with participation extending to leadership roles. This has meant that patient advocates from Africa are in good standing with the international community of patient advocates and can play a strong role in promoting the regulatory infrastructure and management of medicines across Africa (10). Patient advocates are also involved in other global non-profit organizations such as the HTA International (HTAi) Patient and Citizen Involvement in HTA Interest group (35), the Professional Society for Health Economics and Outcomes Research (ISPOR) in health economics and outcomes research (36), and Cochrane for evidence informed healthcare (37). Adoption of civil society involvement provides a mechanism to strengthen the democratic legitimacy of the HTA process, and so decision-making. Such involvement can also lead to better decisions that reflect patients' experiences and values (38). Democratic legitimacy involves questions about where the data for an HTA comes from and how it is analyzed, and prioritizes inclusion rather than scientific rigor. Principles of accountability, fairness, representation, and transparency become important (39).

Stigma attached to diseases is an important aspect for the healthcare of individuals. Patient and community groups have traditionally provided community service delivery models of care, for example in Uganda by the National Organization for People Living with Hepatitis B and Uganda Alliance of Patients' Organizations (34).

Globalization with trade agreements, pharmaceutical company activities and digital technologies contribute to the spread of knowledge about new and promising technologies. This can place pressure on often fragile health systems with limited infrastructure, and frequent absence of government-funded national health insurance schemes. If the State does not have a civil society organization engagement framework set in law, policy, or practice standards, then patient engagement in healthcare decision-making may be largely absent and is a “democratic deficit” (40, 41). Yet mobile technologies and access to the internet mean that African people can be pro-active in monitoring their health and sharing their health information with community health workers and pharmacists to reduce the burden on overstretched, understaffed health centers (42).

It may be important for Africa with its complex societies in terms of gender, tribal aspects, ethnicity, language, etc. to take an active interest in patient preference studies. Research is ongoing to provide understanding of how patient perspectives can be captured with patient preference data for use in decision-making about new medicines and technologies (43–45). Such scientifically-derived quantitative evidence could align with the methodological values of HTA professionals. The European Union Innovative Medicines Initiative (IMI) PREFER project submitted a framework informing objectives, design and conduct, and reporting of patient preference studies to the European Medicines Agency to provide an assessment (46). Its draft opinion states that a case-by-case decision would be needed on the weight put on specific results from such studies (47). The National Institute for Health and Care Excellence (NICE) in the UK undertook a project funded by the patient charity Myeloma UK (2016–2018) and suggested there is a clear scope for better use of quantitative patient preference studies (44).

### Government Initiatives That Have Encouraged Consumer and Community Participation in LMICs

In 2007, Thailand established an autonomous government agency called the National Health Commission Office (NHCO) under the National Health Act. This agency enlists patient groups and civil society groups registered as legal entities and represented in the National Health Commission to follow participatory public policy processes. As an example, when prioritizing services for extension of the UCS benefit package in 2009/2010, civil society, patient groups, and lay people from provincial networks of the National Health Assembly participated. Interventions included setting fees, an HIV/AIDS program, a chronic diseases package and a psychosis package (30).

Thailand has also run a village health volunteer program for decades now to strengthen primary healthcare through health education and self-care support, community, and long-term care (42).

## Possible Ways Forward

In establishing a structure or organization to undertake HTAs, sustainable funding, governance, and accountability are prime factors. Conflicts of interest need to be managed and legitimate processes established (26, 31). People undertaking the HTAs need to have the required skills and competencies, and recommendations need to be implemented. In this perspective we have given an example of where external bodies have helped to establish HTA practices in Ghana. The Thai HTA body founded the active HTAsiaLink Network to undertake knowledge sharing and best practices of HTA in the Asia-Pacific region (48). Network members are also members of INAHTA, with its statement on the importance of patient involvement in HTAs (23).

The IAPO first Virtual African Patients Congress brought IAPO's African patient organization membership together with high-level African healthcare stakeholders including regulators, policy makers and others to “share their vision and experience on how we can build back better African health systems after the pandemic” (10). The African Medicines Agency (AMA), as of November 2021, as a single centralizing regulatory body for medicines in Africa is strongly supported. It is hoped that the activities of the AMA can become patient-centric and that it can build patient engagement into its regulatory framework by applying laws, policies, practices, and standards. Building expertise and capacity is a vital part of the vision of the AMA (49, 50). For some, it is important for medical regulators to involve patients and the public across the spectrum of their work (49, 51). Having patient advocates at the discussion table, as occurred with the IAPO African Patients Congress (9), is an important step in African countries working together to incorporate patient values in healthcare. By being involved, patient and community groups and civil society are acknowledged as having a role in gaining recognition of the AMA and other activities to ensure accessibility to high quality, affordable medicines and to promote democratic legitimacy in setting the health policy agenda.

## Data Availability Statement

The original contributions presented in the study are included in the article, further inquiries can be directed to the corresponding author.

## Author Contributions

All authors listed have made a substantial, direct, and intellectual contribution to the work and approved it for publication.

## Conflict of Interest

The authors declare that the research was conducted in the absence of any commercial or financial relationships that could be construed as a potential conflict of interest.

## Publisher's Note

All claims expressed in this article are solely those of the authors and do not necessarily represent those of their affiliated organizations, or those of the publisher, the editors and the reviewers. Any product that may be evaluated in this article, or claim that may be made by its manufacturer, is not guaranteed or endorsed by the publisher.
